# Stomatal Conductance and Morphology of Arbuscular Mycorrhizal Wheat Plants Response to Elevated CO_2_ and NaCl Stress

**DOI:** 10.3389/fpls.2018.01363

**Published:** 2018-09-19

**Authors:** Xiancan Zhu, Qingjun Cao, Luying Sun, Xiaoqin Yang, Wenying Yang, Hua Zhang

**Affiliations:** ^1^Northeast Institute of Geography and Agroecology, Chinese Academy of Sciences, Changchun, China; ^2^Jilin Academy of Agricultural Sciences, Changchun, China

**Keywords:** carbon isotope discrimination, stomatal aperture, stomatal conductance, stomatal density, water potential

## Abstract

Stomata play a critical role in the regulation of gas exchange between the interior of the leaf and the exterior environment and are affected by environmental and endogenous stimuli. This study aimed to evaluate the effect of the arbuscular mycorrhizal (AM) fungus, *Rhizophagus irregularis*, on the stomatal behavior of wheat (*Triticum aestivum* L.) plants under combination with elevated CO_2_ and NaCl stress. Wheat seedlings were exposed to ambient (400 ppm) or elevated (700 ppm) CO_2_ concentrations and 0, 1, and 2 g kg^−1^ dry soil NaCl treatments for 10 weeks. AM symbiosis increased the leaf area and stomatal density (SD) of the abaxial surface. Stomatal size and the aperture of adaxial and abaxial leaf surfaces were higher in the AM than non-AM plants under elevated CO_2_ and salinity stress. AM plants showed higher stomatal conductance (*g*_*s*_) and maximum rate of *g*_*s*_ to water vapor (*g*_*smax*_) compared with non-AM plants. Moreover, leaf water potential (Ψ) was increased and carbon isotope discrimination (Δ^13^C) was decreased by AM colonization, and both were significantly associated with stomatal conductance. The results suggest that AM symbiosis alters stomatal morphology by changing SD and the size of the guard cells and stomatal pores, thereby improving the stomatal conductance and water relations of wheat leaves under combined elevated CO_2_ and salinity stress.

## Introduction

Arbuscular mycorrhizal (AM) fungi belong to the monophyletic phylum Glomeromycota and form symbiotic associations with the roots of over 80% of land plant species (Smith and Read, 2008). AM fungi (AMF) are obligate biotrophs that have many benefits for the host plants, such as increased nutrient uptake, plant productivity, and abiotic and biotic stress resistance (Zhu et al., 2016a). In turn, the host plants provide the fungi with photosynthetic carbon (Smith and Read, 2008). AM symbiosis has been demonstrated to affect plant performance as it leads to a series of morphological, physiological, biochemical, and molecular changes.

Stomata are formed by two small symmetric guard cells on the epidermis of higher plants that play a central role in the regulation of gas exchange between the inner air space of the leaf and the outer atmosphere (Lawson, 2009). Stomata enable CO_2_ entry into the leaf for photosynthesis while limiting water loss, thus influencing global water and carbon cycles (Blatt et al., 2017). Stomatal conductance (*g*_*s*_) is regulated primary by the aperture of the stomatal pore (SA) and stomatal density (SD), as well as the water transport capacity of the guard cells on the leaf surface (Yan et al., 2012; Huang and Xu, 2015; Gamage et al., 2018). AM symbiosis often alters the stomatal behavior of the host (Augé et al., 2016; Chitarra et al., 2016). *g*_*s*_ differs between AM and non-AM plants; however, the effect of AM on *g*_*s*_ is unpredictable, and its mechanism remains unclear (Smith and Read, 2008; Augé et al., 2015). Changes in *g*_*s*_ are always accompanied by changes in leaf water potential (Ψ) or osmotic adjustment. The influence of AM on *g*_*s*_ may also be associated with altered chemical signals and carbon dynamics of the plant leaves (Ruiz-Lozano and Aroca, 2010; Augé et al., 2015).

Stomatal behavior is modulated by environmental factors, such as elevated atmospheric CO_2_ concentration and salinity stress. To date, the concentration of atmospheric CO_2_ has risen to over 400 μmol l^−1^ (NOAA-ESRL, 2018), and about 7% of the total global land area is occupied by saline soils (Sheng et al., 2008). In the future, global climate change will result in the exacerbation of increasing atmospheric CO_2_ concentration and soil salinization. Therefore, elevated CO_2_ concentration and salinity are important factors that influence plant growth and crop productivity. Major physiological processes are regulated by increased CO_2_ and saline stress, both of which can directly affect gas exchange and water relations (Eller et al., 2014; Yu et al., 2015). Accumulated evidence indicates that *g*_*s*_ is decreased under elevated CO_2_ and salt stress, and elevated CO_2_ improves water use efficiency and Ψ under saline conditions (Pérez-López et al., 2012b; Zaghdoud et al., 2013). However, the underlying mechanisms for the regulation of stomatal formation and development under elevated CO_2_ and salinity remain largely elusive. Studies have documented that the response of SA and SD to environmental conditions are contradictory, implying complexity in the regulation of stomatal morphology and behavior by environmental cues (Yan et al., 2012; Sun et al., 2014).

Importantly, little is known about the effect of AM on plant growth and physiology under combined elevated CO_2_ concentrations and soil salt stress. Some studies have reported that AMF influences the stomatal behavior of leaves exposed to salinity stress or elevated CO_2_ regime. Under salinity stress, AM plants showed higher *g*_*s*_ and lower intercellular CO_2_ concentration and water potential, hereby maintaining a favorable gas exchange capacity and water use efficiency (Sheng et al., 2008; Evelin et al., 2009; Kapoor et al., 2013; Frosi et al., 2018). At elevated CO_2_, the response of *g*_*s*_ to AM symbiosis varies. Syvertsen and Graham (1999) found that the difference in *g*_*s*_ was not significant in AM and non-AM citrus plants grown at elevated CO_2_ concentration. However, Baslam et al. (2012) and Goicoechea et al. (2014) reported that 5-weeks-old AM seedlings of alfalfa had higher *g*_*s*_ when grown at elevated CO_2_ for 2 weeks and lower *g*_*s*_ when grown at elevated CO_2_ for 4 weeks than non-AM plants. Under combined elevated CO_2_ and saline stress, *g*_*s*_ and stomatal morphology in AM and non-AM wheat plants are altered, as reported by Zhu et al. (2016b).

Wheat (*Triticum aestivum* L.) is one of the world's most important cereal crops and grown on about 220 million hectares and yielding more over 715 million tons of grain in 2013 (FAOSTAT, 2015). Our previous study found that AM symbiosis alleviated salinity stress in wheat plants under elevated CO_2_ concentration (Zhu et al., 2016b). In this study, we examined whether AM symbiosis altered the stomatal behavior of wheat plants under elevated CO_2_ concentration and salt stress. To test this, stomatal conductance and stomatal morphology in wheat leaves under different CO_2_ regimes and NaCl levels were determined. In addition, plant growth, leaf water potential, and carbon isotope discrimination (Δ^13^C) were measured to investigate the interaction between water relations and stomatal behavior under combined elevated CO_2_ and salinity stress.

## Materials and methods

### Experimental materials

The AMF (*Rhizophagus irregularis* (Blaszk., Wubet, Renker & Buscot) Sieverd., G.A. Silva & Oehl.) used in this experiment was obtained from INOQ GmbH, Schnega, Germany. The AM inoculum was a mixture of vermiculite with spores, hyphae and root residues (mycorrhiza units, 210 spores per mL substrate).

The soil was sandy loam and collected from the experimental farm of the Department of Plant and Environmental Sciences, Faculty of Science, University of Copenhagen, Taastrup, Denmark. The characteristics of the soil included a pH of 6.8, total C 11.8 g kg^−1^, total N 1.15 g kg^−1^, and Olsen-P 19.2 mg kg^−1^. The soil was sieved by passing through a 2 mm mesh, and then sterilized in the oven at 95°C for 4 h for three consecutive days.

### Experimental design and plant growth

The experiment was conducted with two AM inoculation levels (inoculated with *R. irregularis* and not inoculated as the control), two atmospheric CO_2_ concentrations (ambient, 400 ppm and elevated, 700 ppm CO_2_), and three NaCl levels (0, 1 and 2 g kg^−1^ dry soil) treatments. The experimental pots were 4 L (15.2 cm diameter and 25 cm deep), and the bottom of the pots was covered with mesh (1.5 mm). The pots were filled with 6.0 kg of soil. Twenty mL of AM inoculum was mixed into the soil for the inoculation treatment, and 20 mL sterilized inoculum plus 10 mL AMF-free filtrate from the inoculum suspension was added for the non-inoculation treatment. Half of the pots were then placed into a glasshouse cell with ambient CO_2_ concentration, and the other half were placed into another glasshouse cell with elevated CO_2_ concentration. The CO_2_ concentration in the glasshouse cells was monitored every 6 s by a CO_2_ Transmitter Series GMT220 (Vaisala Group, Helsinki, Finland). Eight seeds of wheat (var. Tuareg) were sown in each pot, and tap water was used for irrigation. The climate conditions in the glasshouse were set at: 25/16 ± 2°C day/night air temperature, 60% relative humidity, 16 h photoperiod and >500 μmol m^−2^ s^−1^ photosynthetic active radiation (PAR) supplied by sunlight plus meta-halide lamps. Three weeks later, the eight seedlings were thinned to four per pot, and 1.0 g N and 0.8 g K per pot were applied to meet the nutrient requirements for plant growth. After 4 weeks, the seedlings were subjected to salinity treatments with NaCl solution. The experiment was a complete randomized block design with four replicates for each treatment.

### Sampling and measurement

Ten weeks after sowing, the *g*_*s*_ of the leaf adaxial and abaxial surfaces was measured by a leaf porometer (Decagon Devices, Inc., Pullman, WA, USA) at 11:00 a.m. Then stomatal morphology was measured according to Zhu et al. (2016b). Briefly, fingernail polish imprints were obtained from the adaxial and abaxial surfaces of the leaf, using clean cellophane tape to transfer the impression to a microscope slide. The imprints were observed by a LEITZ DMRD microscope camera system (Leica Microscope and System GmbH, D 35530, Wetzlar, Germany). Stomatal density (SD), guard cell length (L_g_), guard cell width (W_g_), stomatal pore length (L_p_), and stomatal pore width (W_p_) of the leaf adaxial and abaxial surfaces were measured and stomatal size (SS), stomatal aperture (SA) and anatomical maximum stomatal conductance to water vapor (*g*_*smax*_) were then calculated using the following equation:

$$\begin{array}{lll} SA & = & \pi \times W_{\text{p}}\times L_{\text{p}}/4\\ SS & = & \pi \times W_{\text{g}}\times L_{\text{g}}/4-SA\\ g_{smax} & = & \frac{d\;\text{SD }{\text{a}}_{\max}}{\nu \;\left(\;L_{p}+\;\frac{\pi }{2}\sqrt{\frac{a_{\max}}{\pi }}\right)}, \end{array}$$

where *d* is the diffusivity of water in air (24.6 × 10^−6^ m^2^ s^−1^ at 25°C), a_max_ is the mean maximum stomatal pore area, ν is the molar volume of air (24.4 × 10^−3^ m^3^ mol^−1^ at 25 °C and 101.3 kPa) and π is the mathematical constant (approximated to 3.142) (Franks and Farquhar, 2001).

Leaf area was measured by a leaf area meter (Li-3100, Li-Cor Inc., Lincoln, NB, USA). Leaf Ψ was measured by a pressure chamber (Soil Moisture Equipment Corp., Santa Barbara, CA, USA) on the same leaves (Zhu et al., 2016b).

Leaf samples were oven-dried at 75°C for 3 d and were ground to a fine powder. Plant C and ^13^C concentrations were analyzed using the Dumas dry combustion method in a system consisting of an ANCA-SL Elemental Analyzer coupled to a 20–20 mass spectrometer (Europa Scientific Ltd, Creve, UK). ^13^C composition (δ^13^C) was calculated as:

$$\begin{array}{l} \delta^{13}C=1,000\times \left[\left({\text{R}}_{\text{sample}}-{\text{R}}_{\text{standard}}\right)/{\text{R}}_{\text{standard}}\right], \end{array}$$

where *R*_sample_ is the ^13^C:^12^C ratio of the sample and *R*_standard_ is the ^13^C:^12^C of the Pee Dee Belemnite (PDB) standard. Δ^13^C was calculated as:

$$\begin{array}{l} \Delta^{13}C=\left(\delta_{\text{a}}-\delta_{\text{p}}\right)/\left(1+\delta_{\text{p}}\right), \end{array}$$

where the subscripts a and p refer to air and plant, respectively. The δ_a_ value was taken as −7.7‰ (Yan et al., 2012).

### Statistical analysis

The data were statistically analyzed using Microsoft Excel (Microsoft Corporation, Albuquerque, NM, USA) and SPSS 16.0 (SPSS Inc., Chicago, IL, USA) software. The effects of AMF, CO_2_, and NaCl treatments as well as their interactions on variables were analyzed using analysis of variance (ANOVA). Differences between treatments were considered significant when the *P*-value was less than 0.05 by Tukey's test. Pearson's correlation coefficients between variables were computed in SPSS 16.0.

## Results

### Leaf area

**Figure 1:**
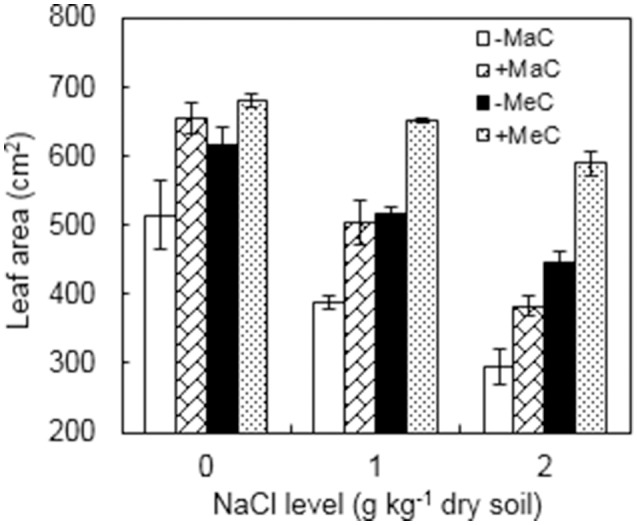
Leaf area of wheat plants inoculated (+M) or not (–M) with *Rhizophagus irregularis* at ambient (aC) and elevated (eC) CO_2_ and 0, 1, and 2 g kg^−1^ dry soil NaCl levels. Vertical bars represent the means ± standard errors.

AM, CO_2_, and NaCl treatments significantly affected the leaf area of the wheat plants (Figure 1, Table 1). Under ambient CO_2_, the leaf area of the AM plants was increased by 27.2, 29.4, and 29.7% at 0, 1, and 2 g kg^−1^ dry soil NaCl levels, and by 10.5, 26.2, and 32.1% under elevated CO_2_ condition, respectively, compared to non-AM plants. Elevated CO_2_ increased but salinity stress decreased leaf area. A significant interactive effect between CO_2_ and NaCl treatments was observed.

**Table 1:** Probabilities of significance for main effects and factor interactions for the variables measured and analyzed using three-way ANOVA.

	**AM**	**CO_2_**	**NaCl**	**AM × CO_2_**	**AM × NaCl**	**CO_2_ × NaCl**	**AM × CO_2_ × NaCl**
Leaf area	**	**	**	ns	ns	**	ns
SD adaxial	ns	*	*	ns	ns	ns	ns
SD abaxial	*	**	**	ns	ns	ns	ns
SS adaxial	ns	*	**	ns	ns	ns	ns
SS abaxial	**	ns	**	ns	*	ns	ns
SA adaxial	**	**	**	ns	ns	**	ns
SA abaxial	**	**	**	ns	ns	**	ns
*g_*s*_* adaxial	**	**	**	ns	ns	ns	ns
*g_*s*_* abaxial	**	**	**	ns	ns	ns	ns
*g_*smax*_* adaxial	**	*	*	ns	ns	ns	ns
*g_*smax*_* abaxial	**	ns	*	ns	ns	ns	*
Δ^13^C	**	**	**	ns	ns	**	ns
Ψ	**	**	**	ns	ns	ns	ns

* P < 0.05;

** *P < 0.01; ns, not significant by Tukey's test*.

### Stomatal morphology

AM, CO_2_, and NaCl treatments significantly affected guard cell length (L_g_) and width (W_g_), and stomatal pore length (L_p_), and width (W_p_) of the wheat leaf adaxial and abaxial surfaces, while adaxial W_g_ was not affected by AM and CO_2_ treatments (Tables 2, 3). In general, elevated CO_2_ and salt stress decreased L_g_, W_g_, L_p_, and W_p_, and AM fungus increased the L_g_, L_p_, and W_p_ of the wheat leaves. There were significant interactive effect of AM × NaCl on abaxial L_g_, CO_2_ × NaCl on W_p_, and AM × CO_2_ × NaCl on abaxial L_g_, W_g_, L_p_, and W_p_.

**Table 2:** Stomatal morphology in the adaxial leaves of wheat plants inoculated (+M) or not inoculated (–M) with *Rhizophagus irregularis* (AM) at CO_2_ and NaCl treatments.

**NaCl (g kg^−1^)**	**CO_2_**	**Inoculation**	**Guard cell length**	**Guard cell width**	**Stomatal pore length**	**Stomatal pore width**
0	Ambient	–M	52.3 ± 0.91	33.8 ± 0.69	37.0 ± 0.66	10.7 ± 0.26
		+M	52.9 ± 2.27	33.8 ± 0.78	39.9 ± 1.94	12.1 ± 0.65
	Elevated	–M	47.3 ± 0.54	32.7 ± 1.05	31.0 ± 0.44	9.21 ± 0.41
		+M	51.3 ± 0.87	33.1 ± 1.16	32.3 ± 0.49	10.2 ± 0.46
1	Ambient	–M	49.6 ± 1.07	28.7 ± 1.87	33.6 ± 0.97	8.51 ± 0.72
		+M	55.6 ± 2.66	30.2 ± 1.59	37.2 ± 2.15	9.97 ± 0.48
	Elevated	–M	45.8 ± 0.43	28.0 ± 0.67	30.8 ± 1.18	9.31 ± 0.30
		+M	50.5 ± 0.40	29.7 ± 0.73	32.0 ± 0.78	9.92 ± 0.20
2	Ambient	–M	45.6 ± 0.79	31.2 ± 2.36	30.2 ± 2.43	9.59 ± 0.67
		+M	49.4 ± 0.51	28.2 ± 1.40	33.8 ± 1.74	9.78 ± 0.56
	Elevated	–M	40.7 ± 0.88	28.3 ± 0.20	26.8 ± 0.81	7.61 ± 0.14
		+M	45.7 ± 0.76	28.0 ± 0.34	30.4 ± 0.53	8.65 ± 0.29
**SOURCE OF VARIATION**						
AM			**	ns	**	**
CO_2_			**	ns	**	**
NaCl			**	**	**	**
AM × CO_2_			ns	ns	ns	ns
AM × NaCl			ns	ns	ns	ns
CO_2_ × NaCl			ns	ns	ns	**
AM × CO_2_ × NaCl			ns	ns	ns	ns

Values are means ± s.e.

** *P < 0.01; ns, not significant by Tukey's test*.

**Table 3:** Stomatal morphology in the abaxial leaves of wheat plants inoculated (+M) or not inoculated (–M) with *Rhizophagus irregularis* (AM) at CO_2_ and NaCl treatments.

**NaCl (g kg^−1^)**	**CO_2_**	**Inoculation**	**Guard cell length**	**Guard cell width**	**Stomatal pore length**	**Stomatal pore width**
0	Ambient	–M	51.5 ± 1.00	31.1 ± 0.19	34.1 ± 1.24	10.1 ± 0.29
		+M	49.5 ± 1.09	34.7 ± 1.07	35.7 ± 1.08	11.6 ± 0.31
	Elevated	–M	44.8 ± 1.19	31.8 ± 0.51	29.3 ± 0.67	8.88 ± 0.26
		+M	51.8 ± 2.95	30.6 ± 1.13	32.3 ± 3.00	8.98 ± 0.48
1	Ambient	–M	45.2 ± 1.61	29.4 ± 1.17	29.3 ± 0.95	9.21 ± 0.31
		+M	54.5 ± 0.50	30.2 ± 1.33	36.8 ± 1.53	9.12 ± 0.28
	Elevated	–M	48.2 ± 0.67	25.7 ± 0.19	32.3 ± 0.77	8.27 ± 0.19
		+M	50.2 ± 0.67	29.6 ± 1.20	32.9 ± 0.73	9.26 ± 0.24
2	Ambient	–M	46.4 ± 0.40	28.1 ± 1.31	30.4 ± 0.48	8.27 ± 0.21
		+M	46.4 ± 0.89	26.0 ± 1.47	32.9 ± 0.56	8.27 ± 0.21
	Elevated	–M	44.0 ± 0.49	26.3 ± 0.44	29.0 ± 0.44	7.42 ± 0.12
		+M	44.8 ± 1.25	28.1 ± 0.27	31.4 ± 0.34	8.17 ± 0.14
**SOURCE OF VARIATION**						
AM			**	*	**	**
CO_2_			*	*	**	**
NaCl			**	**	*	**
AM × CO_2_			ns	ns	ns	ns
AM × NaCl			*	ns	ns	ns
CO_2_ × NaCl			ns	ns	ns	**
AM × CO_2_ × NaCl			**	**	*	**

Values are means ± s.e.

* P < 0.05;

** *P < 0.01; ns, not significant by Tukey's test*.

**Figure 2:**
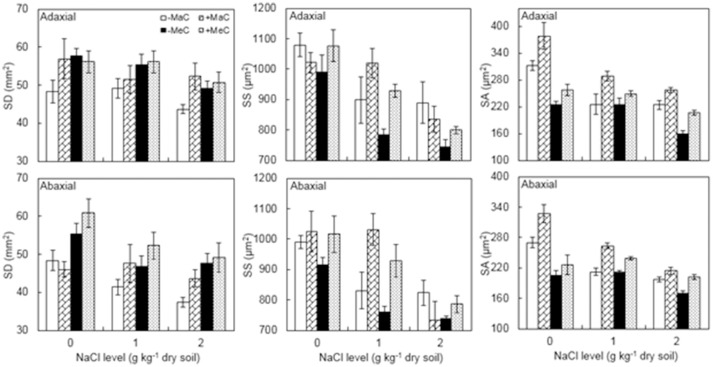
Stomatal density (SD), stomatal size (SS) and stomatal aperture (SA) on the adaxial and abaxial leaf surfaces of wheat plants inoculated (+M) or not (–M) with *Rhizophagus irregularis* at ambient (aC) and elevated (eC) CO_2_ and 0, 1, and 2 g kg^−1^ dry soil NaCl levels. Vertical bars represent the means ± standard errors.

SD, SS, and SA of the leaf adaxial and abaxial surfaces were significantly affected by AM, CO_2_, and NaCl treatments, whereas adaxial SD and SS were not affected by AM and abaxial SS was not affected by CO_2_ (Figure 2, Table 1). Salt stress decreased SD, SS, and SA. AM colonization increased abaxial SD, and adaxial and abaxial SS and SA under elevated CO_2_ and salt stress. Additionally, a significant interactive effect of CO_2_ × NaCl on SA was observed.

### Stomatal conductance

**Figure 3:**
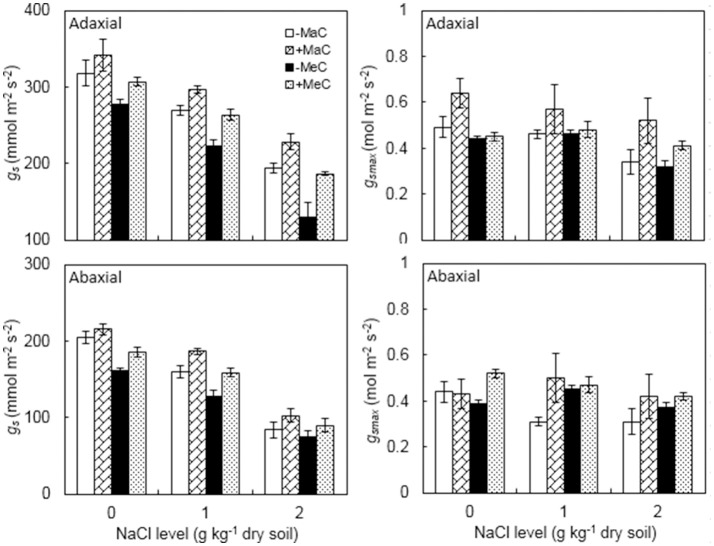
Stomatal conductance (*g*_*s*_) maximum rate of *g*_*s*_ to water vapor (*g*_*smax*_) on the adaxial and abaxial leaf surfaces of wheat plants inoculated (+M) or not (–M) with *Rhizophagus irregularis* at ambient (aC) and elevated (eC) CO_2_ and 0, 1, and 2 g kg^−1^ dry soil NaCl levels. Vertical bars represent the means ± standard errors.

Elevated CO_2_ and salinity significant decreased the *g*_*s*_ of the adaxial and abaxial leaf surfaces (Figure 3, Table 1). However, AM plants had greater *g*_*s*_ than non-AM plants. For adaxial *g*_*s*_, at 400 ppm CO_2_ level, AM colonization increased by 7.5, 10.2, and 17.4% at 0, 1, and 2 g kg^−1^ dry soil NaCl levels, and by 10.6, 18.2, and 43.8% at 700 ppm CO_2_ level. For abaxial *g*_*s*_, at 400 ppm CO_2_ level, AM colonization increased by 5.5, 17.1, and 22.4% at 0, 1, and 2 g kg^−1^ dry soil NaCl levels, and by 15.0, 24.4, and 18.9% at 700 ppm CO_2_ level.

Leaf adaxial and abaxial *g*_*smax*_ were significantly affected by AM, CO_2_, and NaCl treatments, while abaxial *g*_*smax*_ was not affected by CO_2_ (Figure 3, Table 1). There was a significant interactive effect of AM × CO_2_ × NaCl on abaxial *g*_*smax*_. Severe (2 g kg^−1^ dry soil NaCl) salt stress decreased adaxial and abaxial *g*_*smax*_. AM colonization enhanced the *g*_*smax*_ of the adaxial and abaxial surfaces.

### Δ^13^C

**Figure 4:**
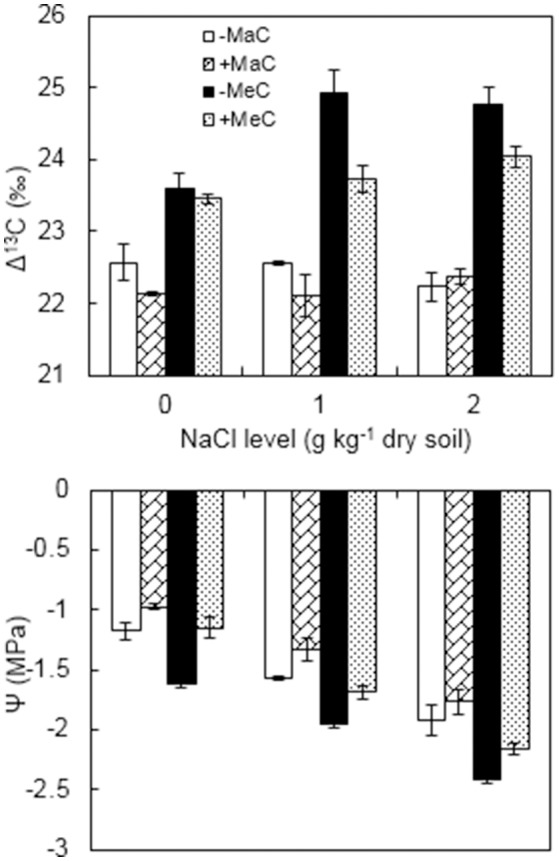
Carbon isotope discrimination (Δ^13^C) and water potential (Ψ) of wheat leaves inoculated (+M) or not (–M) with *Rhizophagus irregularis* at ambient (aC) and elevated (eC) CO_2_ and 0, 1, and 2 g kg^−1^ dry soil NaCl levels. Vertical bars represent the means ± standard errors.

Leaf Δ^13^C was affected by AM, CO_2_, and NaCl treatments (Figure 4, Table 1). Wheat plants had higher Δ^13^C under elevated CO_2_ compared with ambient CO_2_. Under elevated CO_2_ level, salt stress increased Δ^13^C, and AMF decreased Δ^13^C. Moreover, there was a significant interactive effect of CO_2_ × NaCl on Δ^13^C.

### Water potential

Leaf Ψ was affected by AM, CO_2_ and salt treatments (Figure 4, Table 1). Elevated CO_2_ and NaCl stress significantly reduced the Ψ, and AM colonization enhanced the Ψ of the wheat plants. Under ambient CO_2_, the leaf Ψ of the AM plants was enhanced by 16.9, 15.3, and 7.8% at 0, 1, and 2 g kg^−1^ dry soil NaCl levels, and by 29.0, 13.3, and 10.7% under elevated CO_2_ condition, respectively, compared to non-AM plants.

### Relationships between stomatal traits

It was found that the *g*_*s*_ and *g*_*smax*_ of the adaxial and abaxial leaf surfaces were positively correlated with SD, SA, SS, and Ψ (Table 4). Nonetheless, a negative linear relationship between Δ^13^C and *g*_*s*_, *g*_*smax*_, SA, SS, and Ψwas found across all the treatments.

**Table 4:** Pearson's correlation coefficients between stomatal traits.

	**Adaxial *g_*s*_***	**Abaxial *g_*s*_***	**Adaxial *g_*smax*_***	**Abaxial *g_*smax*_***	**Adaxial SD**	**Abaxial SD**	**Adaxial SS**	**Abaxial SS**	**Adaxial SA**	**Abaxial SA**	**Ψ**	**Δ^13^C**
Adaxial *g_*s*_*	1	0.871^**^	0.500^**^	0.317^*^	0.225	0.270	0.696^**^	0.702^**^	0.710^**^	0.760^**^	0.891^**^	−0.507^**^
Abaxial *g_*s*_*		1	0.498^**^	0.363^*^	0.323^*^	0.304^*^	0.657^**^	0.746^**^	0.672^**^	0.751^**^	0.849^**^	−0.400^**^
Adaxial *g_*smax*_*			1	0.264	0.625^**^	0.219	0.123	0.415^**^	0.651^**^	0.517^**^	0.527^**^	−0.314^*^
Abaxial *g_*smax*_*				1	0.265	0.630^**^	0.246	0.186	0.286^*^	0.416^**^	0.238	0.036
Adaxial SD					1	0.502^**^	0.025	0.183	0.227	0.110	0.237	0.146
Abaxial SD						1	0.213	0.308^*^	0.034	0.033	0.200	0.284
Adaxial SS							1	0.481^**^	0.552^**^	0.519^**^	0.741^**^	−0.405^**^
Abaxial SS								1	0.484^**^	0.594^**^	0.709^**^	−0.354^*^
Adaxial SA									1	0.782^**^	0.780^**^	−0.547^**^
Abaxial SA										1	0.710^**^	−0.496^**^
Ψ											1	−0.575^**^
Δ^13^C												1

* P < 0.05;

** *P < 0.01*.

## Discussion

Stomata play a key role in regulating gas exchange on the leaf surface (Lawson, 2009). Plants are constantly faced with environmental stress conditions and have evolved a series of mechanisms to regulate stomatal behavior in response to this stress (Casson and Hetherington, 2010). AM symbiosis has been shown to alter the stomatal behavior of the host plants (Augé et al., 2015). However, the mechanisms underlying the effects of AMF on stomatal traits remain largely elusive, particularly for host plants grown under multiple environmental regimes. Therefore, this study aimed to investigate the effect of AM on *g*_*s*_ and the stomatal morphology of wheat plants under combined elevated CO_2_ and NaCl stress.

In the present study, the *g*_*s*_ of the adaxial and abaxial leaf surfaces in the AM wheat plants was higher than in non-AM plants. AM plants often show higher *g*_*s*_ than non-AM plants under unstressed conditions, elevated CO_2_ concentration and salt stress (Aroca et al., 2013; Goicoechea et al., 2014; Augé et al., 2016; Frosi et al., 2018). A meta-analysis by Augé et al. (2015) indicated that the *g*_*s*_ in AM plants was increased by an average of 24% compared with non-AM plants for sufficiently watered and drought-stressed plants. Moreover, both NaCl stress and elevated CO_2_ decreased *g*_*s*_, which is in agreement with the results by Pérez-López et al. (2012b), Zaghdoud et al. (2013) and Piñero et al. (2014). CO_2_ elevation increased CO_2_ flux into the leaves because the gradient of CO_2_ between the outer and the inner leaf increases (Pérez-López et al., 2012b). Salt stress affected the diffusion of CO_2_ into the leaves due to closed stomata and decreased stomatal and mesophyll conductance (Piñero et al., 2014). Under elevated CO_2_ and NaCl stress, AM plants had higher *g*_*s*_ than non-AM plants, which may be due to decreased photorespiration in AM plants, increased sink strength of AM fungal roots, and a higher capability for CO_2_ assimilation (Aroca et al., 2013; Goicoechea et al., 2014; Zhu et al., 2016b). We also observed a lower reduction in *g*_*s*_ in AM plants compared with non-AM plants with increasing levels of salinity and elevated CO_2_, which suggested that CO_2_ diffusion through the stomata was diminished less, and thus the water status may be better in AM plants (Chen et al., 2017).

Anatomical *g*_*smax*_ represents the maximum rate of *g*_*s*_ to water vapor as calculated by the density, size and geometry of the stomata when fully open (Dow et al., 2014; McElwain et al., 2016). *g*_*smax*_ has been a useful tool for assessing stomatal development with respect to external environmental factors, such as air CO_2_ concentration and salinity (Dow et al., 2014). Our results showed that elevated CO_2_ decreased adaxial *g*_*smax*_, and AMF increased *g*_*smax*_ of both the adaxial and abaxial leaf surfaces. Lammertsma et al. (2011) reported that increased global CO_2_ has led to reduced *g*_*smax*_ in Florida vegetation through SD and a_max_ adaptation within their phenotypic plasticity, which likely represents the adaptation of plants to increased intrinsic water use efficiency via optimizing carbon gain to water loss. *g*_*smax*_, like *g*_*s*_, was greater in AM plants than non-AM plants, which indicated that AM colonization enhanced gas exchange capacity and the potential productivity of the plants. Multiple combinations of increased stomatal density and pore geometries resulted in increased *g*_*smax*_ in AM plants (McElwain et al., 2016). AM was also found to enhance the functioning of the guard cells at maximum turgor pressure or aperture size, thereby increasing gas exchange (Dow and Bergmann, 2014).

In our study, *g*_*s*_ was significantly correlated with SD and SA, which is consistent with the results reported by Kumar et al. (2014) in *Jatropha curcas* grown under elevated CO_2_ condition. Such relationships indicated that both SD and SA control *g*_*s*_ in wheat plants. Franks and Farquhar (2007) suggested several strategies for increasing *g*_*s*_ that were aimed ultimately at increasing the sum of stomatal pore area/depth per unit leaf area, and the simplest and possibly the most accessible option was to increase SD. It is generally accepted that SD is changed by atmospheric CO_2_ concentration, salinity and other environmental factors (Chitarra et al., 2016; Gamage et al., 2018). However, the response of SD to environmental changes is varied and complex. Differences appear to exist among plant species and/or genotypes, experimental facilities, and experimental conditions (Yan et al., 2017). In the present study, NaCl stress decreased SD, and elevated CO_2_ and AM colonization increased SD, which indicated that the response of SD to environmental factors differs. Chitarra et al. (2016) found that SD in AM tomato plants was almost twice that of non-AM plants. Some genes that regulate stomatal development were altered significantly only in the presence of AM symbiosis, and *LeEPFL9* transcript levels were associated with increased SD in AM plants (Chitarra et al., 2016). Moreover, some studies have shown that an increase in SD is not accompanied by a decrease in the size of the stomata under altered environmental conditions (Yan et al., 2012). In contrast, experimental evidence has demonstrated that SD is negatively associated with SS (Lawson and Blatt, 2014). A greater SD associated with smaller stomata was observed, which suggests that dense, small stomatarespond to environmental changes more rapidly, and thus plants reach a high *g*_*s*_ rapidly under favorable conditions and reduce *g*_*s*_ promptly under unfavorable conditions (Hetherington and Woodward, 2003; Drake et al., 2013). AM wheat plants had greater numbers of stomatal compared with non-AM plants, which resulted in higher *g*_*s*_ and better growth under unfavorable conditions.

When plants are exposed to changing environmental conditions for a short period, SA may be the main factor influencing *g*_*s*_, whereas changes in *g*_*s*_ may be determined by the alternation of both SA and SD because SA is more dynamic while SD is more static in response to a changed environment for a long period (Franks and Farquhar, 2007; Yan et al., 2012). SA is regulated by a series of internal signals and environmental cues that serve to “set” stomatal apertures to adapt to the prevailing environmental regimes (Murray et al., 2016). In our plants, as observed in other experiments (Zaghdoud et al., 2013; Zhu et al., 2016b), elevated CO_2_ and NaCl stress caused stomatal closure by affecting the size of the guard cells and stomata. However, AM plants have greater SS and SA than non-AM plants, which indicated that AM symbiosis is able to maintain higher stomatal opening. Therefore, it may be concluded that the increment in *g*_*s*_ of AM wheat plants observed in this study could have been attributed to both increased stomatal number and openings in response to growth at elevated CO_2_ and salt stress. Moreover, SA is primarily determined by the turgor pressure of the guard cells, which is mediated via ion concentration including K^+^, Cl^−^, and Ca^2+^ (Gamage et al., 2018). AM inoculation may have decreased the activity of outward rectifying K^+^ channels relative to that of inward rectifying K^+^ channels, and/or decreased the Cl^−^ release from the guard cells and decreased the Ca^2+^ concentration within them, resulting in stomatal opening (Araújo et al., 2011; Gamage et al., 2018).

The present results showed that NaCl stress and elevated CO_2_ decreased the Ψ of the wheat leaves, which suggests that plants have to reduce their water potential to maintain a continued uptake of water (Pérez-López et al., 2012a). Concurrently, a positive correlation between *g*_*s*_ and Ψ also proved that plants ensure a lower *g*_*s*_ to diminish water losses of the leaf surface. However, our results showed that AM plants had a similar Ψ to non-AM plants under non-stressed conditions, as well as a higher Ψ than non-AM plants under elevated CO_2_ and NaCl stress. The results indicated that under elevated CO_2_ and salinity stress, AM plants are able to maintain better water status and consequently more open stomata and higher *g*_*s*_ compared with their non-AM counterparts. Chitarra et al. (2016) suggested that stomatal closing upon water stress in AM plants is probably not regulated by active ABA-mediated mechanisms but could rather be driven by passive hydraulic-mediated mechanisms. The higher *g*_*s*_ of the AM plants has been associated with a higher Ψ (Augé et al., 2015; Zhu et al., 2016b), and thus the AM symbiosis promotion of leaf hydration would naturally be related to altered stomatal behavior under elevated CO_2_ and salt stress.

Moreover, we noticed a negative linear relationship between *g*_*s*_ and Δ^13^C. Δ^13^C is often used as an indirect evaluation of water use efficiency (Yan et al., 2012). In this study, we confirmed that elevated CO_2_ increased the Δ^13^C of the wheat plants, which was in agreement with the results of Pérez-López et al. (2014). Under elevated CO_2_ concentration, Δ^13^C in the AM plants was lower than the non-AM plants. The Δ^13^C value represents a long-term mean of the intercellular to the atmospheric CO_2_ concentration ratio (Farquhar et al., 1989). Significant differences in Δ^13^C caused by AM, elevated CO_2_ and salinity suggested that these environmental cues have altered the ratio of the intercellular to the atmospheric CO_2_ concentration. Sekiya and Yano (2008) demonstrated that both the increased photosynthetic rate and the decreased SA similarly lower the ratio of the intercellular CO_2_ concentration to the atmospheric CO_2_ concentration, resulting in a reduction in Δ^13^C. Our results showed that Δ^13^C was negatively correlated with SA, which indicated that increased SA was associated with the optimization of leaf gas exchange, such that an increment of water use efficiency (Sun et al., 2014). Similarly, some studies reported that SD was negatively associated with leaf Δ^13^C, indicating that greater SD is associated with higher water use efficiency (Sekiya and Yano, 2008; Yan et al., 2012). Therefore, it is evident that the increment in SA and *g*_*s*_ of the AM wheat plants observed in this study could decrease Δ^13^C and thus improve water use efficiency in response to growth at elevated CO_2_ and salinity stress.

It is well documented that the effect of AM on stomatal behavior is associated with AM-induced changes in plant size (Augé et al., 2015). In this study, leaf area decreased under NaCl stress and increased under elevated CO_2_ concentration. This is consistent with the results that elevated CO_2_ mitigates the detrimental effect of salt stress on plant growth (Geissler et al., 2009; Yu et al., 2015). Under elevated CO_2_ and NaCl stress, the leaf area of AM plants was higher than the non-AM counterparts. A higher leaf area, and thus larger plant size, might affect plant water relations and stomatal behavior. However, according to the meta-analysis by Augé et al. (2015), the effect of AM on stomatal behavior can be independent of the AM promotion of shoot size, although the AM-induced increase in *g*_*s*_ without growth promotion was smaller than the overall AM promotion.

In short, our study showed that AM symbiosis altered the stomatal behavior of wheat plants exposed to elevated CO_2_ concentration and NaCl stress. Compared to non-AM plants, AM wheat plants had higher *g*_*s*_, SD and SA of the adaxial and abaxial leaf surfaces under combined elevated CO_2_ and NaCl stress. Better stomatal conductance and stomatal development allowed the AM plants to optimize stomatal movement and gas exchange, leading to an improvement in water status, as exemplified by the increased Ψ. Our results suggest that the response of stomatal characteristics to environmental changes differs, which poses a significant challenge to the prediction of the effects of climate change on water flux through the stomata (Yan et al., 2017). Our findings also elucidate the effect of AM symbiosis on the stomatal behavior of plants and will be useful in understanding the underlying mechanism of the regulation of stomatal behavior and development by AM symbiosis in response to future climate change scenarios.

## Author contributions

XZ conceived and designed the study; XZ, QC, and LS performed the experiments; XZ, QC, and XY analyzed the data; and XZ wrote the article with contributions by QC, WY, and HZ.

### Conflict of interest statement

The authors declare that the research was conducted in the absence of any commercial or financial relationships that could be construed as a potential conflict of interest.
